# Pilot Plant for the Capture of Ammonia from the Atmosphere of Pig and Poultry Farms Using Gas-Permeable Membrane Technology

**DOI:** 10.3390/membranes11110859

**Published:** 2021-11-07

**Authors:** María Soto-Herranz, Mercedes Sánchez-Báscones, Juan Manuel Antolín-Rodríguez, Pablo Martín-Ramos

**Affiliations:** 1 Departamento de Ciencias Agroforestales, ETSIIAA, Universidad de Valladolid, Avenida de Madrid 44, 34004 Palencia, Spain; mercedes.sanchez@uva.es (M.S.-B.); juanmanuel.antolin@uva.es (J.M.A.-R.); 2 Instituto Universitario de Investigación en Ciencias Ambientales de Aragón (IUCA), EPS, Universidad de Zaragoza, Carretera Cuarte s/n, 22071 Huesca, Spain; pmr@unizar.es

**Keywords:** ammonia capture, livestock housing, gas-permeable membranes, ventilation rate, seasonal variability of ammonia emissions

## Abstract

Gas-permeable membrane (GPM) technology is a possible solution to reduce ammonia (NH_3_) emissions from livestock housing. This paper presents the results obtained with an NH_3_-capture prototype based on the use of expanded polytetrafluoroethylene (ePTFE) membranes in real conditions in a gestating sow house and a free-range laying hen house, comparing them with the results obtained in controlled laboratory conditions for the same type of waste. The NH_3_ present in the air of the livestock housing was captured by reaction with an acidic solution flowing inside the membranes. The periods of continuous operation of the pilot plant were 232 days at the pig farm and 256 days at the poultry farm. The NH_3_ recovery rate at the end of those periods was 2.3 and 0.4 g TAN·m^−2^·d^−1^ in the pig and the poultry farms, respectively. The limiting factor for the capture process was the NH_3_ concentration in the air, with the highest recovery occurring in the most concentrated atmosphere. Differences in NH_3_ capture were observed between seasons and farms, with capture efficiencies of 1.62 and 0.33 g·m^−2^·d^−1^ in summer and 3.85 and 1.20 g·m^−2^·d^−1^ in winter for pig and poultry farms, respectively. The observed differences were mainly due to the higher ventilation frequency in the summer months, which resulted in a lower NH_3_ concentration inside the houses compared to the winter months. This is especially important when considering the real applicability of this technology. The results obtained suggest that GPM technology holds promise for limiting NH_3_ emissions from livestock housing with NH_3_ ambient concentrations close to 20 ppm or as part of manure storage facilities, given that it allows for recovery of nitrogen in a stable and concentrated solution, which can be used as a fertilizer.

## 1. Introduction

Ammonia (NH_3_) emissions from agriculture and intensive livestock farming have been a source of public concern since the 1980s, as NH_3_ is a precursor of particulate matter formation and a source of acidification and eutrophication in ecosystems [1,2,3,4,5]. In addition, it produces odors that can cause a nuisance in nearby population centers [6,7,8].

The livestock sector is estimated to be responsible for 78% of biodiversity loss, 80% of soil acidification and air pollution, 81% of global warming, and 73% of water pollution [9]. The contribution of poultry and pig farms to total NH_3_ emissions is significant (around 20%) due to the large number of animals. Total NH_3_ emissions from the European pig sector are estimated at 606 kt N·year^−1^, those associated with poultry meat production amount to 217 kt N·year^−1^, and total emissions associated with egg production amount to 88 kt N·year^−1^ [1]. 

Ammonia emissions occur at all stages of manure management. Ammonia nitrogen from livestock excrement is the main source of NH_3_. In barns, NH_3_ volatilizes from the manure, spreads through the building, and is finally removed by the ventilation system. Factors such as temperature, ventilation rate, humidity, stocking density, litter quality, and feed composition (crude protein) can affect NH_3_ levels [1]. 

In order to reduce these emissions, environmental legislation has been developed in the European Union (EU) to cut down on pollution from this sector. The Integrated Pollution Prevention and Control Directive [10] obliges EU farmers to apply best available techniques (BATs), which encompass optimized emission reduction techniques. However, many of these techniques require significant capital investment and can sometimes promote undesirable cross-pollutant effects [1]. Furthermore, limiting emission reductions through the application of BATs alone may be insufficient to meet environmental goals [11]. 

For this reason, the EU, through its Directive 2016/2284 [12], has established emission ceilings for all member states since 2010 with the aim of mitigating air pollution. Specifically, emission ceilings were proposed for 2020 and 2030, as well as intermediate levels for 2025, which would allow emissions to be controlled in order to reach the 2030 target. In the case of NH_3_, for Spain, emissions in 2030 under the baseline scenario are estimated to reach 453 kt, exceeding the established ceiling of 353 kt. This level of emissions would be 6% lower than the inventoried data for 2005 (483 kt). Therefore, the forecasts for compliance with the emission ceiling targets are negative and would entail sanctions. 

In addition to the incorporation of measures that contribute to the mitigation of NH_3_ emissions in livestock housing and an improvement in manure management and waste treatment, the application of technologies that help to reduce NH_3_ emissions in livestock facilities while allowing N recovery is of particular importance.

Recently, new technologies such as simultaneous nitrification–denitrification [13,14], struvite precipitation [15,16], and ammonia removal have been applied to remove total ammonia nitrogen (TAN) from wastewater [17,18] or to transform it into other non-polluting compounds, thus allowing its recovery and reuse. Studies focusing on the recovery of total ammonia nitrogen (TAN) from different sources, such as chicken manure, pig manure, anaerobically digested slurry, or digested chicken manure, have shown that the gas-permeable membrane (GPM) technique is very effective for the recovery of NH_3_, reducing the concentration of TAN in the emission sources in a short period of time [19,20,21,22,23]. Furthermore, this method can be used both to remove NH_3_ from slurry before it escapes into the air and to recover volatilized NH_3_ directly from the air [24,25].

The main advantages of this technology over traditionally used technologies such as reverse osmosis [26], adsorption with zeolites by ion exchange [27], or the use of adsorption towers [28] for the treatment of NH_3_ emissions in livestock facilities are that it has a low energy consumption, requires a low working pressure, does not require effluent pre-treatment, does not need the addition of any alkaline reagents [21,29], and does not drastically disrupt on-farm operation.

The ammonia capture process in GPM technology involves the flow of NH_3_ through a gas-permeable microporous membrane by diffusion and the subsequent recovery of NH_3_ in an acidic solution on the other side of the membrane. The NH_3_ combines with H^+^ to form non-volatile ammonium ions (NH_4_^+^), transforming them into an ammonium salt that can be used as a fertilizer. This fertilizer can be exported to nitrogen-deficient regions, avoiding soil and air pollution problems in the producing areas.

The European LIFE+ project “Ammonia Trapping,” in which this research is framed, aims to contribute to the reduction of NH_3_ emissions generated by livestock waste produced in pig and poultry farms through the application of GPM technology. As part of the project, two NH_3_ capture prototypes have been designed, built, and tested in real conditions, one aimed at direct recovery from the slurry (liquid-recovery prototype) and the other from the air inside the livestock housing (gas-recovery prototype). This paper presents the results on NH_3_ capture from the air of two animal houses (pig and poultry farms) obtained at pilot scale with GPM technology using the latter prototype, comparing them with those obtained for the same type of livestock waste under laboratory conditions.

## 2. Materials and Methods

### 2.1. Location

**Figure 1:**
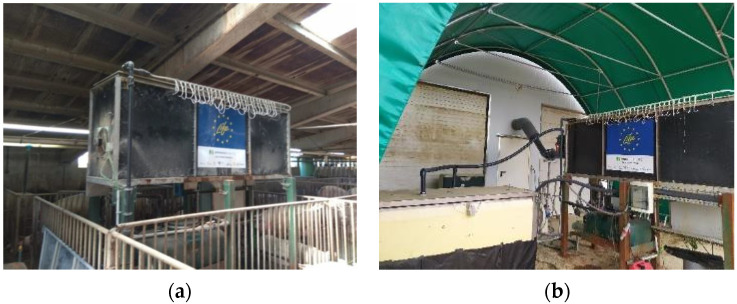
Location of the prototype: (**a**) inside the gestating sow house, (**b**) connected outside the free-range laying hen farm.

The pilot-scale gas-recovery prototype was installed in two locations (Figure 1): inside a gestating sow house with 912 places, with a natural ventilation system, in Guardo (Palencia, Spain), and outside a free-range laying hen house with 8350 places, with a natural ventilation system, in Aldealafuente (Soria, Spain).

### 2.2. Composition of Livestock Waste

The chemical characterizations of raw pig slurry and poultry manure are presented in Table 1.

### 2.3. Characteristics of the e-PTFE Membrane

Gas-permeable tubing was made of ePTFE (Zeus Industrial Products Inc., Orangeburg, SC, USA), with an outer diameter of 5.2 mm, a wall thickness of 0.64 mm, a polymer density of 0.95 g·cm^−3^, a porosity < 60%, an average pore size length of 12.7 ± 5.9 µm, and an average pore size width of 1.3 ± 0.9 µm. The pores of the ePTFE membrane were elongated in the extrusion process.

**Figure 2:**
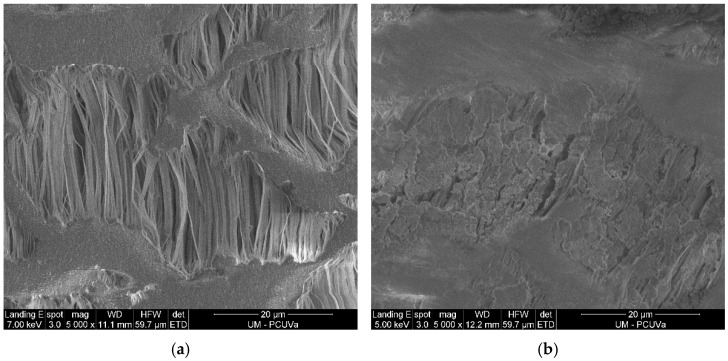
Scanning electron microscopy (SEM) images of the inner surface of (**a**) new and (**b**) used ePTFE membrane samples. Images were taken at 5000× magnification and the scale bar equals 20 μm in length. Micrograph (**a**) shows typical elongated pore structures of different sizes, whereas micrograph (**b**) shows the salt crystals formed with use adhered to the pore structures.

The morphology of the inner surface of new and used membrane samples (Figure 2) was analyzed by scanning electron microscopy (SEM) at the Advanced Microscopy Unit of the University of Valladolid. SEM images were obtained using a FEI QUANTA 200F device (FEI Company, Hillsboro, OR, USA).

### 2.4. Components of the Pilot-Scale Gas-Recovery Prototype

**Figure 3:**
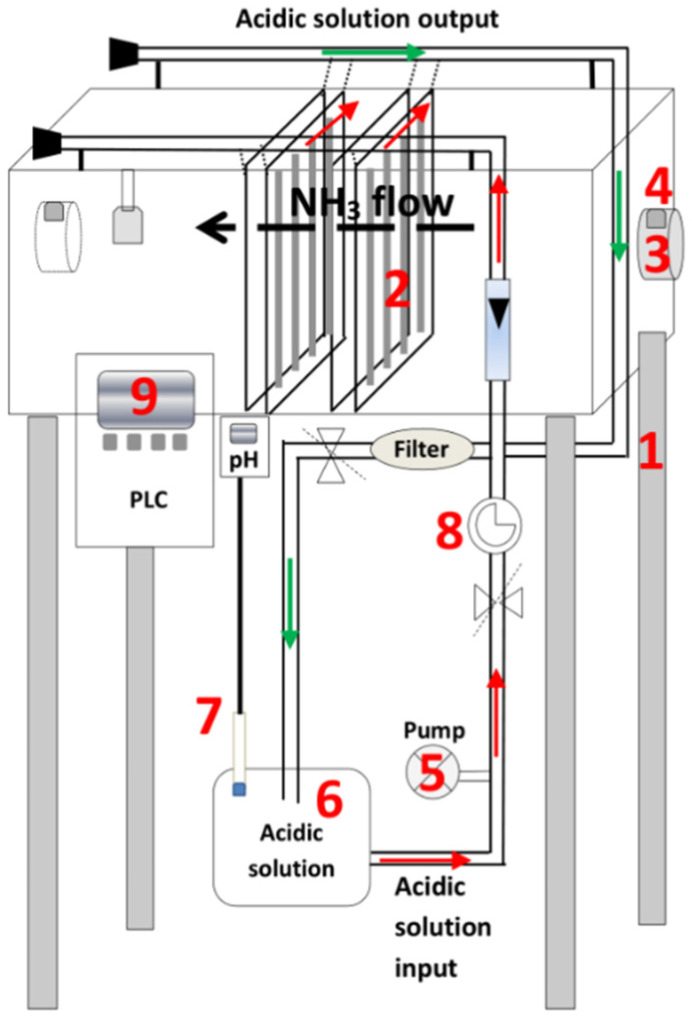
Schematic of the gas-capture prototype.

The gas-recovery prototype consists of several elements, which are shown in Figure 3, labelled with the following numbers:

1. A 2.6 m^3^ steel structure.
2. Thirty-two membrane panels arranged vertically within the steel structure. Each panel contains 14.8 m of e-PTFE tubular membrane (ZEUS, Orangeburg, SC, USA) attached by plastic connections to a plastic support net (1 cm mesh), attached to stainless steel frames (0.750 m × 0.495 m). The surface area of the membrane used is 7.7 m^2^.
3. One 150 W single-phase wall-mounted fan.
4. Two DURTOX IP65-v07 ammonia sensors (Duran^®^ Electrónica, Madrid, Spain).
5. A 0.56 kW acidic solution recirculation pump. A flow rate of 2.1 L·h^−1^ was used.
6. A 0.25 m^3^ tank for the storage of the acidic solution. Specifically, the capture solution rises to a sealed distribution pipe connected to the 32 parallel membrane panels. Another pipe collects the acidic capture solution from the membranes of the 32 panels, returning it to the storage tank by gravity.
7. pH and temperature probes.
8. A pressure gauge to monitor the pressure of the acidic solution. This is an important control parameter, given that a low pressure can impede the circulation of the liquid through the membranes, slowing down the capture rate, whereas a high pressure can cause damage to the membranes. The pressure selected to lead the capture solution to the sealed distribution tube is 0.2 bar, with 0.5 bar being the highest admissible outlet pressure to avoid damaging the membranes used.
9. A PLC system (Siemens, Munich, Germany) was used to control the equipment. It generates a continuous record of the temperature and pH of the acidic solution, keeping the latter below 2 to favor NH_3_ capture.

### 2.5. Equipment Used at Laboratory Scale

A detailed description of the equipment used at laboratory scale was presented in a previous article [30]. The experimental design consisted of 11 L airtight chambers, into which 1 L of pig slurry or 500 g of poultry manure were introduced to recover the emitted NH_3_ gas through a GPM, using the method developed by Szogi, et al. [31]. In both cases, 10 mg∙L^−1^ of allylthiourea (98%) was added to the residue as a nitrification inhibitor. A nitrification inhibitor is needed because nitrification would result in NH_3_ oxidation and in a reduction in the pH of the waste material, which would negatively affect the overall NH_4_ recovery efficiency.

The acidic solution reservoir used to capture the ammonia contained 1 L of TAN capture solution (1 N H_2_SO_4_). This solution was continuously recirculated into the membrane using a pulsating pump (Pumpdrive 5001, Heidolph, Schwabach, Germany). The flow rate used, as in the pilot-scale prototype, was 2.1 L·h^−1^. 

An ePTFE tube, identical to the one used in the pilot-scale prototype, with a membrane length of 1 m, suspended in the chamber above the slurry or manure, was used for NH_3_ capture.

### 2.6. Operating and Monitoring Procedure

The pilot-scale prototype was evaluated over an approximately eight-month period in each type of livestock housing between 2019 and 2020. In both cases, a two-month trial was first conducted to optimize the performance of the equipment, after which it was operated in continuous mode. At each site, a single long-term experiment was conducted to evaluate the performance of the gas-capture prototype under real environmental conditions. In the case of the laboratory-scale gas prototype, it was operated for 60 days, which was sufficient to determine the maximum TAN capture achieved. A single experiment was carried out, using poultry slurry or manure as the TAN emission source.

In the case of the pilot-scale prototype, air from inside the housing was extracted by a wall-mounted fan at a flow rate 52.6 m^3^·h^−1^ and directed into the prototype structure. A total of 150 L of 1 N H_2_SO_4_ was used as a capture solution to concentrate the nitrogen, which was uninterruptedly circulated through the membranes over the entire duration of the experiment. The pH of the capture solution was kept below 2, since at a pH above 2 the available H^+^ ions would not be sufficient to continuously react with NH_3_, which would slow down the capture rate. For this purpose, concentrated H_2_SO_4_ (96–98%, Panreac, Glenview, IL, USA) was manually added to the capture solution until a pH < 1 was obtained each time the pH of the solution increased above 2.

### 2.7. Sampling and Analysis

The pilot-scale tests at both sites were conducted in continuous operation, i.e., by continuously introducing air from inside the vessel into the prototype and maintaining the same acidic trapping solution for the duration of the experiments. Throughout the experimental period, 3–4 samples of acidic solution were collected weekly and kept refrigerated at 4 °C until they were analyzed. Analyses of pH, temperature, EC, and total ammoniacal nitrogen (NH_4_-N or TAN) were performed. Electrical conductivity and pH were measured with a Crison Basic 20 pH meter (Crison Instrumentos S.A., Barcelona, Spain). Total nitrogen analysis—measured as TAN—was performed by distillation (with a Kjeltec^TM^ 8100 nitrogen distillation unit; Foss Iberia S.A., Barcelona, Spain) by uptake of the distillate in borate buffer and subsequent titration with 0.2 mol·L^−1^ HCl [32].

Throughout the experimental period, data collected by the NH_3_ sensors of the prototype were downloaded and collected and compared with static measurements inside the vessels, carried out with a portable Draegër X-700 device (Lubeca, Germany). 

The same parameters were monitored at laboratory scale.

### 2.8. Calculations

The mass of TAN removed (expressed in mg TAN) was determined as the difference between the amount of TAN at the beginning and at the end of the experiment in slurry/manure. The mass of TAN recovered (expressed in mg TAN) was determined by the amount of TAN captured at the end of the experiment in the acidic solution. The TAN removal efficiency (%) was estimated by dividing the mass recovered by the removed mass.

The TAN mass flux (J, expressed in g TAN·cm^−2^·d^−1^), which occurs as a consequence of the gas concentration rate across the membrane [33], was determined by considering the mass of TAN captured per day and the GPM pipe surface area.

## 3. Results and Discussion

### 3.1. Process Parameters

#### pH and Electrical Conductivity Values

To better understand the behavior of these variables during the experimental period on the farms, the reaction that occurs upon contact of NH_3_ (gas) with the acidic capture solution is shown in Equation (1).

2NH_3_ + H_2_SO_4_ → (NH_4_)_2_SO_4_ (1)

**Figure 4:**
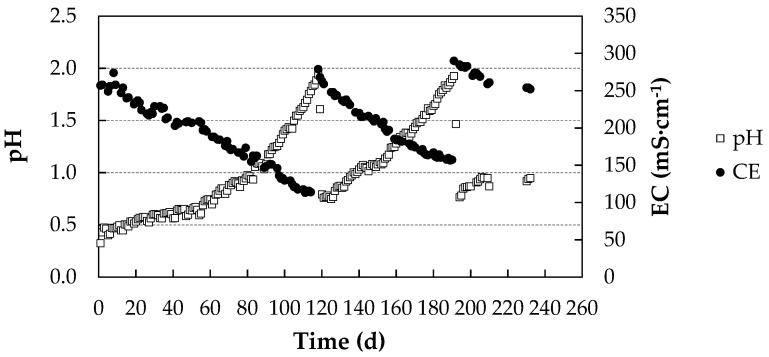
pH and electrical conductivity (EC) values of the acidic capture solution during the experimental period at the pig farm.

Figure 4 shows the pH and electrical conductivity (EC) values of the acidic capture solution in the pig farm during the experimental period (232 days). pH corrections had to be made on days 118 and 191 of the process. 

The capture of NH_3_ by the acidic solution generated a reduction in conductivity and an increase in pH due to the reaction of NH_3_ with the free protons of H_2_SO_4_. This indicates that the capture process progressed optimally, as the concentration of (NH_4_)_2_SO_4_ in the solution increased and the medium was alkalinized by the reduction of the amount of H^+^ ions present in the solution. The contribution to the electrical conductivity values was lower in (NH_4_)_2_SO_4_ than in H_2_SO_4_, so the electrical conductivity was reduced.

The evolution of the electrical conductivity of the acid uptake solution fitted well to a straight line in each of the three sections depicted in Figure 3 (R^2^ = 0.9786; R^2^ = 0.9699; R^2^ = 0.9454). 

The uptake of NH_3_ depends on its ambient concentration, so higher concentrations lead to a faster variation of pH and EC [23]. In our experiment, the ambient concentration was kept below 20 ppm according to legislation [34], which implies lower NH_3_ uptake and milder pH variations.

**Figure 5:**
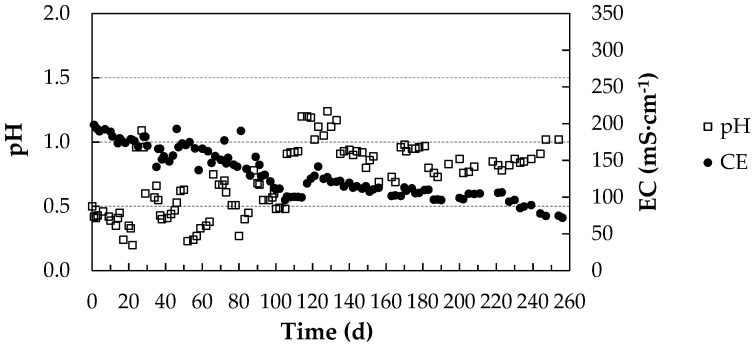
pH and EC values of the acidic solution during the experimental period at the poultry farm.3.1.2. Evolution of Total Ammoniacal Nitrogen (TAN) Captured in the Acidic Solution.

Figure 5 shows the evolution of pH and EC in the acidic trapping solution in the poultry farm during the experimental period (256 days). As in the previous case, an inverse relationship between both parameters was observed. Upon comparison with Figure 4, it was observed that the pH and EC variations in the acidic solution were milder than in the pig farm, influenced by a lower NH_3_ uptake due to lower environmental concentrations of NH_3_, ranging from 3 to 9 µL NH_3_∙L^−1^.

**Figure 6:**
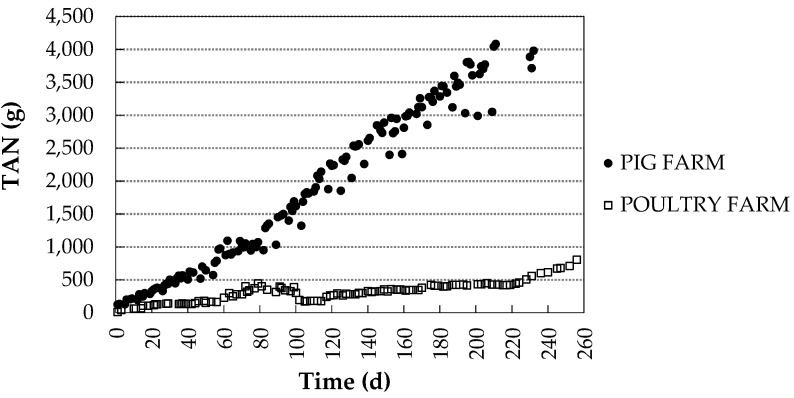
Evolution of the amount of TAN captured in the acidic solution during the experimental period in each farm.

The evolution of the amount of TAN captured in the acidic solution during the experimental period in the two farms is shown in Figure 6. 

The final concentration of TAN in the acidic solution in the pig farm was 28.22 ± 0.04 g TAN∙L^−1^, hence capturing 4108 g TAN. The average daily recovery obtained during the whole period was 17.7 g TAN·d^−1^, with a TAN recovery rate at the end of the period of 2.29 g TAN∙m^−2^∙d^−1^. The capture process was linear, proportional to time (R^2^ = 0.9886). 

The final concentration of TAN in the acidic solution in the poultry farm was 5.40 ± 0.04 g TAN∙L^−1^, capturing 794 g TAN. The average daily recovery obtained during the whole period was 3.14 g TAN·d^−1^, with a TAN recovery rate at the end of the period of 0.41 g TAN∙m^−2^∙d^−1^. The capture process was also proportional to time (R^2^ = 0.9886).

The results obtained with the atmospheric ammonia capture prototype showed a significantly higher ammonia capture (five times higher) in the pig farm than in the poultry farm. This can be explained, as indicated above, by the higher ambient NH_3_ concentration in the pig farm (*ca*. 20 µL NH_3_∙L^−1^) than in the poultry farm (in the 3–9 µL NH_3_∙L^−1^ range). This result is surprising, given that the nitrogen concentration in poultry manure is much higher than in pig slurry, but it can be easily justified by the differences in environmental management between the two farms: Although ventilation in both farms was natural (opening of windows), in the poultry farm, sluicegates were also opened to allow the hens to go outside for several hours a day, remaining open during this period of time and significantly reducing the environmental concentration of NH_3_ (and therefore its capture). 

### 3.2. Differences in TAN Capture between Seasons

The amount of TAN recovered, the TAN capture rate per day, and the TAN mass flux per day and membrane surface according to season and farm type are summarized in Table 2. The period (30 days) selected as representative of the summer season for the pig farm was from 9 August 2018 to 7 September 2018, whereas the winter period selected was from 4 December 2018 to 2 January 2019. The average outdoor ambient temperature in the summer month was 17.9 °C and 5.8 °C in the winter month. The selected period in the summer season for the poultry farm was from 23 August 2019 to 23 September 2019, and the winter period was from 8 November 2019 to 8 December 2019, with average outdoor ambient temperatures of 18.2 °C and 5.2 °C, respectively.

The results show differences in TAN capture between the summer and winter seasons and between farm types (explained above). A higher TAN capture in winter was observed in both farms. The TAN capture efficiency obtained in winter was more than twice as high as in summer in the case of the pig farm, and more than three times as high in the case of the poultry farm. This result is consistent with a higher concentration of NH_3_ in the atmosphere of the livestock houses in winter than in summer, a period when it is necessary to ventilate much more frequently due to the high temperatures and the concentration of harmful gases present inside the livestock housing [35,36,37]. 

These observations are interesting, as they raise the possibility of establishing the operation of the gas-capture prototype in alternating periods depending on the natural ventilation cycles of the livestock housing, being more useful in the winter months. Similarly, it would be possible to automatically run the system during the hours in which the building’s natural ventilation decreases. In fact, there are numerous studies that indicate variations in NH_3_ emissions between day and night, showing a greater diurnal trend [38,39,40,41]. This diurnal pattern responds directly to indoor temperature and inversely to ventilation rate [37,38,42]. 

Possible adaptations of the system to prevent NH_3_ from being discharged into the atmosphere in the summer by the frequent ventilation are proposed below (see Section 3.6).

### 3.3. Correlation between TAN Concentration in the Acidic Solution and Electrical Conductivity

**Figure 7:**
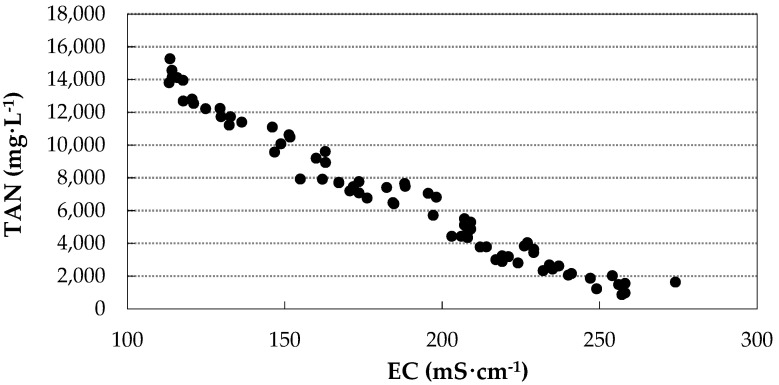
TAN concentration values versus EC of the acidic solution for the pig farm experiment up to the first pH correction.

To investigate the existence of a correlation between TAN and EC variables, only the data from the pig farm up to the first pH correction were selected. The values of TAN concentration versus EC are shown in Figure 7. 

To determine the relationship between the variables, Pearson’s correlation coefficient was applied and a value of −0.982 was obtained, indicating a strong correlation (inverse relationship) and therefore a strong dependence between the two variables. These results are in agreement with those found by other authors [43,44], who observed a significant linear relationship between EC and slurry nutrients (total nitrogen, ammoniacal nitrogen, and potassium). When the acidic solution has a high concentration of dissolved salts, there are fewer free ions available to react with NH_3_, so the EC values are lower.

These results point to the possibility of estimating the concentration of NH_3_ present in the capture solution in situ in a simple and indirect way through the measurement of the EC of the solution, avoiding the need to carry out the TAN analysis in the laboratory.

### 3.4. Estimation of Laboratory TAN Recovery Balances for Each Type of Waste

Ammonia capture performance at the farm level is difficult to assess due to variations in NH_3_ concentration in the environment resulting from management operations (ventilation, door opening, etc.). Therefore, these values were characterized at laboratory scale under controlled conditions over a period of 60 days, using pig slurry and poultry manure as TAN emission sources. The data for the calculation of the nitrogen mass balances recovered at laboratory scale are summarized in Table 3.

The values for TAN recovered, average daily capture, and mass flow at laboratory scale were higher with poultry manure than with pig slurry. This is because in the absence of the ventilation differences mentioned for the farms, the higher initial TAN content in poultry manure compared to pig slurry (6.41 vs. 1.91 g TAN) leads to a higher NH_3_ concentration in the airtight chamber and thus to higher NH_3_ uptake in the acidic solution.

The results of the laboratory-scale experiments indicate a recovery efficiency of 73.3% of TAN removal when using pig slurry as the emission source and of 98.4% when using poultry manure. 

### 3.5. Analysis of TAN Recovery Results

#### 3.5.1. Comparison with TAN Recovery Results Reported in the Literature

A comparison of the TAN recovery results reported herein with those obtained in other recent studies, either at laboratory or at pilot scale, in which GPM membranes have been used to recover NH_3_ from livestock waste (either from the air—suspended systems— or directly from the waste—submerged systems—), is presented in Table 4.

Concerning pilot-scale experiments, Rothrock et al. [25] studied the recovery of ammonia from 32.5 kg of poultry litter and obtained a higher recovery efficiency (10.4–28.6 g·m^−2^·d^−1^) despite using a membrane surface area 20 times smaller than in this study. This result can be explained by the high NH_3_ concentration present in the atmosphere of that experiment, up to 100 times higher than the highest concentration recorded in the farm in this study (915 vs. 9 µL∙L^−1^). No direct comparisons can be made with the work by Buabeng et al. [45] in terms of recovery rate or recovery efficiency, but they obtained 4.08 g TAN·L^−1^ of acidic solution (vs. 5.3 g TAN·L^−1^ of acidic solution in this work). With regard to swine slurry, Molinuevo-Salces et al. [23] recently carried out a pilot-scale study using microporous ePTFE membranes with the same characteristics as in this study for the removal of ammonia directly from manure. Those authors achieved average TAN recovery values in 5 batches of approximately 3111 g TAN in only 20 days of experiment (vs. 4108 g TAN in 232 days in this study). This difference can be explained by the type of installation of the GPM system: By immersing the membrane directly in the slurry, given that the concentration of NH_3_ in the residue is higher than that present in the air, they were able to achieve a recovery of the same order as that achieved in this study, but in a shorter period of time. If the capture efficiencies per membrane surface area are compared, the values attained for the submerged system (with a 13 m^2^ surface area) are over 8 times higher than for the suspended one (19.7 vs. 2.3 g·m^−2^·d^−1^).

In relation to laboratory-scale experiments, for the poultry waste, the ammonia recovery rate and recovery efficiency were higher than those reported by other authors (Table 4). In the case of Rothrock et al. [25], this may be tentatively ascribed to the use of flat ePTFE membranes instead of tubular ones. Conversely, for swine slurry (or anaerobically digested liquid swine manure), the TAN recovery rate and capture efficiency were lower than those reported by other authors [20,22], who used submerged GPM systems. This is explained by the fact that the NH_3_ concentration contained in slurry/manure is much higher than its concentration in the environment.

Compared to a previous study by our group [30] conducted at laboratory scale in which artificial solutions were used as a nitrogen-emission source at TAN concentrations of 3000 (comparable to pig slurry) and 6000 mg·L^−1^ (comparable to poultry manure), higher values of TAN mass have been recovered, but lower values in terms of recovery rate have now been obtained: For artificial solutions of 3000 mg TAN·L^−1^, TAN recovery rates of 6–7 g TAN∙m^−2^∙d^−1^ were previously achieved, whereas for solutions of 6000 mg TAN·L^−1^, rates of up to 21. 4 g TAN∙m^−2^∙d^−1^ were found. The fact that those recovery rates were higher than the ones reported here can be ascribed to the use of artificial solutions composed of NH_4_Cl + NaHCO_3_, which contribute to high NH_3_ emissions by shifting the ammonium–ammonia equilibrium towards NH_3_ formation in a basic medium while minimizing the data variability associated with livestock waste.

#### 3.5.2. Comparison between Farm and Laboratory-Scale Results

Comparing the values obtained under controlled laboratory conditions with the values obtained under real farm conditions, it was observed that the TAN capture values were much higher at the farm for both types of waste: At the pig farm, capture values of 17.7 g·d^−1^ were obtained compared to 0.022 g·d^−1^ at the laboratory with pig slurry, and at the poultry farm, 3.14 g·d^−1^ were obtained compared to 0.084 g·d^−1^ at the laboratory with poultry manure. These differences mainly result from the different membrane surfaces of the capture equipment used (7.7 m^2^ of membrane for the pilot-scale prototype versus 163.4 cm^2^ for the laboratory equipment). However, it is also important to consider that waste is constantly produced on the farm, which generates continuous emissions and maintains the NH_3_ concentration in the air, thus favoring its capture, whereas in the laboratory the NH_3_ source is gradually depleted as the experiments progress.

Comparing the mass fluxes, i.e., eliminating the difference in membrane surfaces, similar values were observed for pig slurry at laboratory and farm scale: 1.4 and 2.3 g TAN∙m^−2^∙d^−1^, respectively. In the case of poultry manure, the results at farm level were much lower than those obtained in the laboratory: 0.41 versus 5.1 g TAN∙m^−2^∙d^−1^, respectively. Such differences must be related, as mentioned above, to the ventilation of the hen housing, which resulted in a low ambient NH_3_ concentration, making its uptake difficult. In addition, straw litter was present on the farm, which absorbed the excrement, further reducing NH_3_ volatilization in comparison to laboratory conditions, in which no straw litter was used.

### 3.6. Applicability of the Tested Ammonia Trapping System and Future Work

In view of the results obtained, the installation of the NH_3_-trapping system in livestock houses is only effective under conditions of high NH_3_ concentrations in the environment. Taking into account that current animal welfare legislation (e.g., Directive 2007/43/EC [46], which lays down minimum measures for the protection of chickens kept for meat production) sets 20 ppm NH_3_ as the permissible limit for environmental parameters, it seems more appropriate to install the NH_3_ capture system in livestock facilities with forced ventilation systems so that the outflow waste air is cleaned by the GPM-based system before its release into the environment, or in facilities with natural ventilation in winter periods (when ventilation is lower). It could also be applied as a complement to covered slurry/manure storage systems. In this sense, the reference techniques for reducing NH_3_ emissions, identified in the UN Economic and Social Council’s “Framework Code of Good Agricultural Practice for the Reduction of Ammonia Emissions (ECE/EB.AIR/120)” of 7 February 2014 [47], include the storage of slurry/manure using solid covers (“airtight” cover, roof or tent-like structure), which is an optimal context for the application of NH_3_ capture systems like the one tested here.

Such covered waste storage pits would be a particularly interesting solution for farms with natural ventilation systems like the ones studied herein, which can easily prevent NH_3_ from being discharged into the atmosphere in the summer by the frequent ventilation by implementing daily evacuation of the waste to the storage pits, to which the NH_3_ recovery system would be connected.

The system is currently being tested on a pig farm with a forced ventilation system, where it has been connected to a gas extraction chimney stack, and it is being evaluated at laboratory scale in a covered slurry storage system. In the near future, it will be installed at farm scale in covered slurry storage ponds to evaluate its performance at full scale.

## 4. Conclusions

This study provides pilot-scale data on the suitability of GPM technology for NH_3_ capture from livestock-housing air. The average daily TAN capture values for the naturally ventilated pig and poultry farms studied were 17.7 and 3.14 g·d^−1^, respectively, with recovery rates per membrane area of 2.3 and 0.41 g TAN·m^−2^·d^−1^, respectively. A comparison with the results of experiments carried out under controlled conditions at laboratory scale, in which TAN recovery efficiencies of 73.3 and 98.4% and mass flow rates of 1.4 and 5.1 g TAN·m^−2^·d^−1^ were achieved when using slurry and poultry manure as emission sources, respectively, points to better performance of the capture system at the pig farm. The low NH_3_ concentration inside the free-range laying hen house (3–9 ppm compared to 20 ppm in the gestating sow house), due to better ventilation, was a limiting factor in achieving good NH_3_ capture yields. The effect of ventilation also led to significant seasonal variations in NH_3_ uptake, which was significantly higher in winter months (3.85/1.62 and 1.20/0.33 g TAN·m^−2^·d^−1^ in winter/summer for the pig and poultry farms, respectively). Thus, the tested technology may be particularly suitable for pig farms during the winter months, for livestock houses with forced ventilation systems, or coupled to biodigesters or slurry ponds with solid covers.

## Figures and Tables

**Table 1:** Chemical characterization of pig slurry and poultry manure.

Parameters *	Pig Slurry	Poultry Manure
H (%) ^a^	84.7 ± 0.3	15.9 ± 0.2
OM (%) ^b^	63.5 ± 0.7	75.2 ± 0.7
C/N ^b^	8.70	9.70
pH ^a^	8.17 ± 0.02	8.88 ± 0.00
EC (mS·cm^−1^) ^a^	1154 ± 5.0	5.58 ± 0.16
TN (mg·L^−1^) ^b^	6446 ± 202	10,723 ± 1710
NH_4_-N (mg·L^−1^) ^a^	2524 ± 147	663 ± 69
P_2_O_5_ (mg·L^−1^) ^b^	8674 ± 243	12,329 ± 670
K (mg·kg^−1^) ^b^	51,984 ± 13,614	12,784 ± 463
Na (mg·kg^−1^) ^b^	9567 ± 3853	1510 ± 57
Ca (mg·kg^−1^) ^b^	9958 ± 162	40,115 ± 1665
Mg (mg·kg^−1^) ^b^	7464 ± 165	4046 ± 120
Cu (mg·kg^−1^) ^b^	124 ± 5	24 ± 1
Fe (mg·kg^−1^) ^b^	962 ± 36	550 ± 17
Mn (mg·kg^−1^) ^b^	213 ± 7	178 ± 8
Zn (mg·kg^−1^) ^b^	369 ± 11	189 ± 11
Cr (mg·kg^−1^) ^b^	4.42 ± 0.75	3.59 ± 0.21
Ni (mg·kg^−1^) ^b^	8.10 ± 0.33	3.89 ± 0.30
Pb (mg·kg^−1^) ^b^	1.60 ± 0.05	0.75 ± 0.32
Cd (mg·kg^−1^) ^b^	0.16 ± 0.01	0.25 ± 0.02
Hg (µg·kg^−1^) ^b^	0.24 ± 0.09	2.8 ± 0.05

* Moisture content (H), organic matter (OM), electrical conductivity (EC), total nitrogen (TN), ammoniacal nitrogen (NH_4_-N), total phosphorus (P_2_O_5_). Values are expressed as mean ± standard deviation (n = 3). ^a^ Measurements based on wet weight; ^b^ measurements based on dry weight.

**Table 2:** Acidic solution TAN uptake data representative of one summer and one winter month for the two types of livestock farms studied.

Parameters	Pig Farm		Poultry Farm	
	Summer	Winter	Summer	Winter
Mass of TAN recovered (g)	376	893	82.4	371.2
Daily average TAN capture rate (g·d^−1^)	38	185	8.6	12.7
TAN capture efficiency (g TAN·m^−2^·d^−1^)	1.62	3.85	0.33	1.20

**Table 3:** Mass balance of nitrogen (measured as TAN) recovered at laboratory scale using slurry and poultry manure.

Type of Waste	TAN Initial Mass (g)	TAN Mass Removed (g)	TAN Mass Recovered (g)	Average Daily Capture (g·d^−1^)	Mass Flow (g·m^−2^·d^−1^)
Pig slurry	1.91	1.78	1.31	0.022	1.4
Poultry manure	6.41	5.12	5.04	0.084	5.1

**Table 4:** Comparison of results attained in pilot scale and laboratory scale reported in the literature.

Scale	Substrate	Type of Membrane Mounting	Ammonia Recovered (g)	Ammonia Recovery Rate (g·m^−2^·d^−1^)	Recovery Efficiency (%)	Reference
Pilot	Poultry litter	Suspended ePTFE	794	0.41	−	This study
			29.94–48.83	10.42–28.63	97.7–100	[25]
			4.08	−	−	[45]
	Swine slurry	Suspended ePTFE	4108	2.29	−	This study
		Submerged ePTFE	3111	19.72	62.03	[23]
Laboratory	Poultry litter	Suspended ePTFE	5.04	5.1	98.4	This study
			0.240	1.37	89.9	[24]
			0.107	1.25	88.4	[25]
	Synthetic solution (6000 mg·L^−1^ TAN)		2.993	13.0	96.4	[30]
	Liquid fraction of digested chicken manure	Submerged PDMS	0.088–0.110	1.22–1.48	−	[19]
	Swine slurry	Suspended ePTFE	1.31	1.4	73.3	This study
	Synthetic solution (3000 mg·L^−1^ TAN)		1.609	7.0	97.2	[30]
	Anaerobically digested liquid swine manure	Submerged ePTFE	1.442–2.936	2.65–6.05	76–95	[20]
	Swine slurry		2.280	3.92	99	[22]

PDMS = polydimethylsiloxane.

## Data Availability

The data presented in this study are available on request from the corresponding author. The data are not publicly available due to their relevance as part of an ongoing Ph.D. thesis.

## References

[1] Giner Santonja G., Georgitzikis K., Scalet B.M., Montobbio P., Roudier S., Sancho L.D. (2017). Best Avaible Techniques Reference Document for the Intensive Rearing of Poultry or Pigs.

[2] Ndegwa P.M., Hristov A.N., Arogo J., Sheffield R.E. (2008). A review of ammonia emission mitigation techniques for concentrated animal feeding operations. Biosyst. Eng..

[3] Erisman J.W. (2004). The Nanjing declaration on management of reactive nitrogen. Bioscience.

[4] Sutton M.A., Erisman J.W., Dentener F., Möller D. (2008). Ammonia in the environment: From ancient times to the present. Environ. Pollut..

[5] Aneja V.P., Roelle P.A., Murray G.C., Southerland J., Erisman J.W., Fowler D., Asman W.A.H., Patni N. (2001). Atmospheric nitrogen compounds. II: Emissions, transport, transformation, deposition and assessment. Atmos. Environ..

[6] Schiffman S.S. (1998). Livestock Odors: Implications for Human Health and Well-Being. J. Anim. Sci..

[7] Wing S., Horton R.A., Marshall S.W., Thu K., Tajik M., Schinasi L., Schiffman S.S. (2008). Air pollution and odor in communities near industrial swine operations. Environ. Health Perspect..

[8] Donham K.J., Wing S., Osterberg D., Flora J.L., Hodne C., Thu K.M., Thorne P.S. (2007). Community health and socioeconomic issues surrounding concentrated animal feeding operations. Environ. Health Perspect..

[9] Leip A., Billen G., Garnier J., Grizzetti B., Lassaletta L., Reis S., Simpson D., Sutton M.A., De Vries W., Weiss F. (2015). Impacts of European livestock production: Nitrogen, sulphur, phosphorus and greenhouse gas emissions, land-use, water eutrophication and biodiversity. Environ. Res. Lett..

[10] European Union (2017). Decisión de Ejecución (UE) 2017/302 de la Comisión, de 15 de febrero de 2017, por la que se establecen las conclusiones sobre las mejores técnicas disponibles (MTD) en el marco de la Directiva 2010/75/UE del Parlamento Europeo y del Consejo respecto a la cría intensiva de aves de corral o de cerdos. Off. J. Eur. Union L.

[11] Loyon L., Burton C.H., Misselbrook T., Webb J., Philippe F.X., Aguilar M., Doreau M., Hassouna M., Veldkamp T., Dourmad J.Y. (2016). Best available technology for European livestock farms: Availability, effectiveness and uptake. J. Environ. Manag..

[12] EC—European Commission (2016). Directive (EU) 2016/2284 of the European Parlament and of the Council of 14 December 2016 on the reduction of national emissions of certain atmospheric polluTANts, amending Directive 2003/35/EC and repealing Directive 2001/81/EC. Off. J. Eur. Commun..

[13] Yang S., Yang F. (2011). Nitrogen removal via short-cut simultaneous nitrification and denitrification in an intermittently aerated moving bed membrane bioreactor. J. Hazard. Mater..

[14] Zhang D., Lu P., Long T., Verstraete W. (2005). The integration of methanogensis with simultaneous nitrification and denitrification in a membrane bioreactor. Process Biochem..

[15] Huang H., Zhang P., Xiao J., Xiao D., Gao F. (2017). Repeatedly using the decomposition product of struvite by ultrasound stripping to remove ammonia nitrogen from landfill leachate. Ultrason. Sonochem..

[16] He S., Zhang Y., Yang M., Du W., Harada H. (2007). Repeated use of MAP decomposition residues for the removal of high ammonium concentration from landfill leachate. Chemosphere.

[17] Zangeneh A., Sabzalipour S., Takdatsan A., Yengejeh R.J., Khafaie M.A. (2021). Ammonia removal form municipal wastewater by air stripping process: An experimental study. South African J. Chem. Eng..

[18] Chen T.L., Chen L.H., Lin Y.J., Yu C.P., Ma H.-w., Chiang P.C. (2021). Advanced ammonia nitrogen removal and recovery technology using electrokinetic and stripping process towards a sustainable nitrogen cycle: A review. J. Clean. Prod..

[19] Sürmeli R.Ö., Bayrakdar A., Çalli B. (2018). Ammonia recovery from chicken manure digestate using polydimethylsiloxane membrane contactor. J. Clean. Prod..

[20] Dube P., Vanotti M., Szogi A., González M.C.G. (2016). Enhancing recovery of ammonia from swine manure anaerobic digester effluent using gas-permeable membrane technology. Waste Manag..

[21] Daguerre-Martini S., Vanotti M.B., Rodriguez-Pastor M., Rosal A., Moral R. (2018). Nitrogen recovery from wastewater using gas-permeable membranes: Impact of inorganic carbon content and natural organic matter. Water Res..

[22] Garcia-González M.C., Vanotti M.B. (2015). Recovery of ammonia from swine manure using gas-permeable membranes: Effect of waste strength and pH. Waste Manag..

[23] Molinuevo-Salces B., Riaño B., Vanotti M.B., Hernández-González D., García-González M.C. (2020). Pilot-scale demonstration of membrane-based nitrogen recovery from swine manure. Membranes.

[24] Rothrock M.J., Szögi A.A., Vanotti M.B. (2010). Recovery of ammonia from poultry litter using gas-permeable membranes. Trans. ASABE.

[25] Rothrock M.J., Szögi A.A., Vanotti M.B. (2013). Recovery of ammonia from poultry litter using flat gas permeable membranes. Waste Manag..

[26] Masse L., Massé D.I., Pellerin Y., Dubreuil J. (2010). Osmotic pressure and substrate resistance during the concentration of manure nutrients by reverse osmosis membranes. J. Memb. Sci..

[27] Milan Z., Sánchez E., Weiland P., De Las Pozas C., Borja R., Mayari R., Rovirosa N. (1997). Ammonia removal from anaerobically treated piggery manure by ion exchange in columns packed with homoionic zeolite. Chem. Eng. J..

[28] Bonmatí A., Flotats X. (2003). Air stripping of ammonia from pig slurry: Characterisation and feasibility as a pre- or post-treatment to mesophilic anaerobic digestion. Waste Manag..

[29] Zarebska A., Romero Nieto D., Christensen K.V., Fjerbæk Søtoft L., Norddahl B. (2015). Ammonium fertilizers production from manure: A critical review. Crit. Rev. Environ. Sci. Technol..

[30] Soto-Herranz M., Sánchez-Báscones M., Antolín-Rodríguez J.M., Vanotti M.B., Martín-Ramos P. (2021). Effect of acid flow rate, membrane surface area, and capture solution on the effectiveness of suspended gpm systems to recover ammonia. Membranes.

[31] Szogi A.A., Vanotti M.B., Rothrock M.J. (2014). Gaseous Ammonia Removal System. U.S. Patent.

[32] Rice E.W., Baird R.B., Eaton A.D. (2017). Standard Methods for the Examination of Water and Wastewater.

[33] Blet V., Pons M.-N., Greffe J. (1989). Separation of ammonia with a gas-permeable tubular membrane. Anal. Chim. Acta.

[34] Villalba T. (2021). Código de Protección y Bienestar Animal. https://boe.es/biblioteca_juridica/codigos/codigo.php?id=204&modo=2&nota=0.

[35] Tabase R.K., Millet S., Brusselman E., Ampe B., De Cuyper C., Sonck B., Demeyer P. (2020). Effect of ventilation control settings on ammonia and odour emissions from a pig rearing building. Biosyst. Eng..

[36] Philippe F.X., Cabaraux J.F., Nicks B. (2011). Ammonia emissions from pig houses: Influencing factors and mitigation techniques. Agric. Ecosyst. Environ..

[37] Ni J.Q., Liu S., Diehl C.A., Lim T.T., Bogan B.W., Chen L., Chai L., Wang K., Heber A.J. (2017). Emission factors and characteristics of ammonia, hydrogen sulfide, carbon dioxide, and particulate matter at two high-rise layer hen houses. Atmos. Environ..

[38] Wang-Li L., Li Q.F., Wang K., Bogan B.W., Ni J.Q., Cortus E.L., Heber A.J. (2013). The national air emissions monitoring study’s Southeast Layer Site: Part I. Site characteristics and monitoring methodology. Trans. ASABE.

[39] Fairchild B.D., Czarick M., Harper L.A., Worley J.W., Ritz C.W., Hale B.D., Naeher L.P. (2009). Ammonia concentrations downstream of broiler operations. J. Appl. Poult. Res..

[40] Guarino M., Claudio F., Navarotto P., Valli L., Mascatelli G., Rossetti M., Mazzotta V. (2003). Ammonia, methane and nitrous oxide emissions and particulate matter concentrations in two different buildings for fattening pigs. Proc. Int. Symp. Gaseous Odour Emiss. from Anim. Prod. Facil..

[41] Aarnink A.J.A., Wagemans M.J.M. (1997). Volatilización de amoniaco y concentración de polvo afectado por los sistemas de ventilación de las naves de engorde de cerdos. Transacciones de la ASAE.

[42] Hayes M.D., Xin H., Li H., Shepherd T.A., Zhao Y., Stinn J.P. (2013). Ammonia, greenhouse gas, and particulate matter emissions of aviary layer houses in the Midwestern U.S. Trans. ASABE.

[43] Moral R., Perez-Murcia M.D., Perez-Espinosa A., Moreno-Caselles J., Paredes C. (2005). Estimation of nutrient values of pig slurries in Southeast Spain using easily determined properties. Waste Manag..

[44] Provolo G., Martínez-Suller L. (2007). In situ determination of slurry nutrient content by electrical conductivity. Bioresour. Technol..

[45] Buabeng F., Hashem F.M., Millner P., Matias B.V., Timmons J., Arthur A. (2018). Controlling poultry house ammonia emmissions using gas permeable membrane systems. Br. J. Environ. Sci..

[46] (2007). Diario de la Unión Europea Diario de la Unión Europea. Diario Oficial de la Unión Europea L.

[47] (2014). Guidance Document on Preventing and Abating Ammonia Emissions from Agricultural Sources; ECE/EB.AIR/120, United Nations, Economic and Social Council, Economis Commission for Europe, Executive Body for the Convention on Long-range Transoundary Air Polution, 100 s. https://www.unece.org/fileadmin/DAM/env/documents/2012/EB/ECE_EB.AIR_120_ENG.pdf.

